# Climate resilient and environmentally sustainable radiology: a framework for implementation

**DOI:** 10.1093/radadv/umaf014

**Published:** 2025-04-02

**Authors:** Chloe DesRoche, Felipe Castillo, Sonali Sharma, Beth Zigmund, Julian Dobranowski, Myles Sergeant, Linda Varangu, Kate Hanneman

**Affiliations:** Department of Diagnostic Radiology, Queens University, Kingston, ON, K7L 2V7, Canada; Department of Medical Imaging, University of Toronto, Toronto, ON, M5T 1W7, Canada; Joint Department of Medical Imaging, University Medical Imaging Toronto, University Health Network (UHN) and Sinai Health System (SHS), Toronto, ON, M5G 2C4, Canada; Department of Radiology, Faculty of Medicine, University of British Columbia, Vancouver, V5Z 1M9, Canada; Department of Radiology, Larner College of Medicine at the University of Vermont Medical Center, Burlington, VT, 05405, USA; Department of Radiology, McMaster University, Hamilton, ON, L8S 4L8, Canada; Faculty of Health Sciences, McMaster University, Hamilton, ON, L8S 4L8, Canada; The Canadian Coalition for Green Health Care, Verdun, QC, H4H 1W4, Canada; Department of Medical Imaging, University of Toronto, Toronto, ON, M5T 1W7, Canada; Joint Department of Medical Imaging, University Medical Imaging Toronto, University Health Network (UHN) and Sinai Health System (SHS), Toronto, ON, M5G 2C4, Canada

**Keywords:** climate change, environmental sustainability, radiology, medical imaging, sustainability

## Abstract

Climate change adversely impacts human health and transformations in our approach to work are needed to build environmentally sustainable and climate resilient radiology systems. Radiology practices must reduce greenhouse gas emissions generated in the delivery of care while simultaneously building infrastructure and processes to anticipate, respond to, and recover from climate-related environmental events. The purpose of this review is to highlight the links between climate change, human health, and radiology; discuss mitigation, adaptation, and response approaches; describe opportunities to leverage existing knowledge such as pandemic planning and supply chain management; and develop a radiology resilience checklist to assess vulnerabilities and inform actions necessary to achieve environmentally sustainable and climate resilient practices. The proposed framework is based on 5 pillars of climate resilience capacity—threshold, coping, recovery, adaptive, and transformative. Key actions include increasing awareness of the health impacts of climate change, optimizing infrastructure, improving supply chain management, reducing energy use, and addressing health disparities through collaboration with stakeholders. These strategies are needed to reduce the environmental impact radiology service delivery, prepare for and minimize the effects of climate change on imaging departments, and build capacity to recover quickly from climate-related environmental impacts, ultimately improving planetary health and human well-being.

**Abbreviations**

GHG = Greenhouse gas; USD = United States dollar; IT = information technology; COVID-19 = Coronavirus disease 2019.

**Summary**

To build climate resilient and environmentally sustainable radiology systems and build infrastructure to anticipate, and recover from climate-related events, radiologists must engage and work with stakeholders within and outside our profession.

**Essentials**

- Urgent action and collaboration are needed to build climate resilient and environmentally sustainable radiology systems.
- To achieve this transformation, strategies are needed in radiology including mitigation (reducing greenhouse gas emissions), adaptation (managing the impacts of climate change), and resilience (enduring, recovering, and improving from disruptions).
- A Radiology Resiliency Checklist can be used to assess vulnerabilities and inform actions guided by 5 pillars of climate resilience—threshold, coping, recovery, adaptive, and transformative capacity.

## Introduction

Human activities, primarily burning fossil fuels and deforestation, increase greenhouse gas (GHG) emissions, leading to climate change and global warming. Resulting environmental disruptions affect the atmosphere, oceans, and land, posing a major global threat to human health and quality of life.^1^ The concept of planetary health has emerged as a solutions-oriented field focused on understanding and addressing the impacts of destabilized environmental systems on all life on Earth. Protecting the planet’s health is essential to sustain human health, well-being, and survival.

Environmental changes have disrupted the global climate system, resulting in biodiversity loss, increased pollution, and resource scarcity.^2^ These are complex interconnected phenomena that ultimately affect every aspect of human health. Globally, substantial morbidity and mortality is caused by environmental disruptions such as heatwaves, floods, wildfires, and storms.^3^ Climate change also results in indirect health impacts such as mental health challenges arising from stress, loss of housing, forced relocation, economic instability, and conflict.^4^

Given the profound impact of climate change on human health, urgent transformation of healthcare—including radiology—is needed to build climate resilient and environmentally sustainable systems. To fulfill this transformation, radiology practices must reduce GHG emissions generated in the delivery of care while simultaneously building infrastructure and processes to anticipate, prepare for, and recover from climate-related environmental events.

The purpose of this review is to highlight the links between climate change, human health, and radiology; discuss mitigation, adaptation, and resilience climate response approaches; describe opportunities to leverage existing knowledge such as pandemic planning and supply chain management; and develop a radiology resilience checklist to assess vulnerabilities and inform actions necessary to achieve environmentally sustainable and climate resilient practices.

## Impact of climate change on health systems and radiology

Climate change and related environmental exposures impact human health, increasing the severity, frequency, and mortality of multiple diseases.^5^ Virtually all organ systems are affected by climate change, with extreme temperatures and air pollution associated with increased cardiovascular and stroke mortality,^6^ pneumonia and lung cancer,^7^ dementia,^8^ psychiatric disorders,^9^ autoimmune disease,^10^ renal disease,^11^ and gastrointestinal illness.^12^ Changes in temperature, rainfall, and humidity affect the transmission of many infectious diseases including water and vector borne illnesses.^13^

Climate change-related environmental exposures are also associated with increased healthcare utilization including emergency department visits, hospital admissions, and medical imaging utilization.^14–16^ A nationwide US study reported a 7.8% increase in emergency department visits for any cause on days of extreme heat.^15^ In an analysis of 1 666 420 medical imaging studies from 4 emergency departments over a 10-year period, Hanneman et al^17^ found that short-term exposures to ambient heat and fine particulate air pollution (PM_2.5_) were associated with overall imaging utilization increases of 5.1% and 4.0%, respectively. When stratified by imaging modality, both heat exposure days and air pollution exposure days were associated with increased utilization of radiography and computed tomography (CT) but not US or magnetic resonance imaging (MRI).^17^ Increased utilization of imaging associated with ambient heat was a function of higher emergency department patient volumes, as expected.^15^ Increased imaging utilization associated with higher short-term air pollution exposure was a function of both increased patient volumes and increased per-patient imaging, suggesting that patient presentations associated with exposure to heat and poor air quality may differ. Increased medical imaging associated with climate-related environmental exposures is relevant with respect to preparedness planning and the excess GHG emissions generated by increased imaging.^18^ Excess medical imaging attributed to heat and air pollution across Canada generates excess GHG emissions of 36 261 and 40 648 kg CO_2_e/year, respectively, exacerbating the environmental impact of delivery of radiology services in a negative feedback loop.^19^

Climate change also increases the cost of healthcare. A study of 10 climate-sensitive events in the United States in 2012 reported 20 568 hospitalizations and 17 857 emergency department visits.^20^ Morbidity costs to the health system were $1.6 billion, which rose to $10 billion when mortality costs were included. Over the past decade, average annual economic losses from weather-related extreme events have increased by 23%,^21^ with global economic losses due to climate-related health impacts projected to reach $12.5 trillion by 2050, according to the World Economic Forum.^22^ A recent study in China estimated that annual hospitalizations due to heatwaves contributed to an excess economic burden of 5 billion yuan (approximately USD$700 million) per year.^23^ In radiology, further research is needed to estimate the direct and indirect financial costs of climate change on radiology departments, including damage to infrastructure and workforce shortages.

The effects of climate change on human health are not distributed equally. There are significant disparities, with the largest effects in already vulnerable groups and communities. For example, 92% of pollution-related deaths in 2017 occurred in low- and middle-income countries.^24^ In the United States, 72% of low-income neighborhoods experience elevated heat exposures during the day.^25^ On an individual level, climate change disproportionately affects health at extreme ages (children and older adults) and people who work outdoors, as these groups are more exposed and vulnerable to the effects of extreme temperatures, pollution, and vector-born infectious diseases.^26^ Climate change is a social determinant of health, and targeted actions can mitigate its disproportionate effects on vulnerable groups. Inclusion of climate justice into global health strategies is essential to address these inequities.

## Leverage existing knowledge

Existing knowledge gained from other fields and prior experiences can be the foundation for climate resilient and sustainable radiology practices. One of the timelier examples is the coronavirus disease 2019 (COVID-19) pandemic, which provided lessons relevant to climate preparedness, such as disaster planning protocols and supply chain management. Key lessons that can be applied to building climate resilience in radiology include the ability for large scale and global behavior change during the COVID-19 pandemic. Individuals, organizations, and governments demonstrated the willingness and capacity to make profound changes to public life such as lockdowns and other interventions to limit the spread of disease. The societal response to these interventions can potentially be used to inspire large-scale changes to promote environmental sustainability across healthcare.

Despite similarities in the COVID-19 pandemic and climate change as 2 global crises warranting urgent, large-scale collective action, there are also striking differences in responses. Behavior change to limit climate change has historically been slow in contrast to the speed associated with behavior change to stop the spread of COVID-19. Meijers et al^27^ investigated reasons for the contrast between behavioral responses to the COVID-19 pandemic and the climate crisis. They found government policy was a critical driver of global behavioral change during the pandemic, suggesting that stronger government response to the climate crisis may stimulate widespread, climate-friendly behavioral change. They also found that the perception of “close others” being susceptible to climate threats, as well as communicating the necessity of individual actions to combating climate change, were potential means of spurring behavioral change. These results emphasize the need for government policies and regulations to ensure that delivery of healthcare and radiology services are environmentally sustainable, as well as education on the human health effects of climate change and the cumulative effect of changes in individual radiology departments to accelerate sustainable behaviors. Importantly, broad changes are needed including collaboration with industry partners to ensure supply chains and the entire lifecycle of imaging products and equipment are sustainable, from raw material extraction to manufacturing, packaging, transportation, installation, and end-of-life.

Unequal health outcomes associated with the COVID-19 pandemic also parallel health disparities associated with climate change.^28^ Vulnerability to climate change is determined by exposure to environmental hazards along with the capacity to respond to them.^29^ Individuals who have typically contributed the least to global GHG emissions are often impacted the most. Radiologists can implement outreach programs, prioritize access to care, and develop policies that ensure affordable medical imaging services in low- and middle-income countries globally and in underserved communities within higher-income countries where disparities in the distribution of resources exist.^30^ Actions to ensure equitable access to medical imaging require capacity building globally while minimizing the detrimental environmental effects of the delivery of radiology services.

The pandemic also exposed vulnerabilities in global supply chains. In radiology, supply chain disruptions resulted in a sudden critical shortage of iodine-based contrast media. This unexpected shortage forced departments to conserve resources, prioritize indications, triage patients based on risk, and explore alternative imaging strategies.^31^ Medical imaging companies were also compelled to establish strategies to manage the contrast media life cycle better and to work with radiology departments to reduce contrast dose delivery, reduce waste and recycling of unused contrast. Climate change has already resulted in similar supply chain failures; for example, the intravenous saline shortages occurring in the wake of hurricanes Maria and Helene.^32^ Preparing for potential future related events, radiology departments should implement robust supply chain management systems and consider diversifying suppliers to maintain operations better. Radiology departments can enhance their operational resilience by integrating lessons from the pandemic with proactive strategies aimed at improving environmental sustainability and health equity.

Finally, information from routine data sources can be used to support informed decision making to optimize resources (including financial, human, and supplies) and strengthen resilience. By integrating climate and weather data, radiology departments can prepare for shifts in disease severity, incidence and volume of medical imaging requests. For example, an integrated dashboard with emergency department patient registration volumes and local climate data on air quality and temperature could alert radiology leaders to potential surges in CT requests, triggering deployment or re-allocation of radiologists and technologists before a backlog develops.^17^

## Climate response pathways in radiology

**Figure 1.**
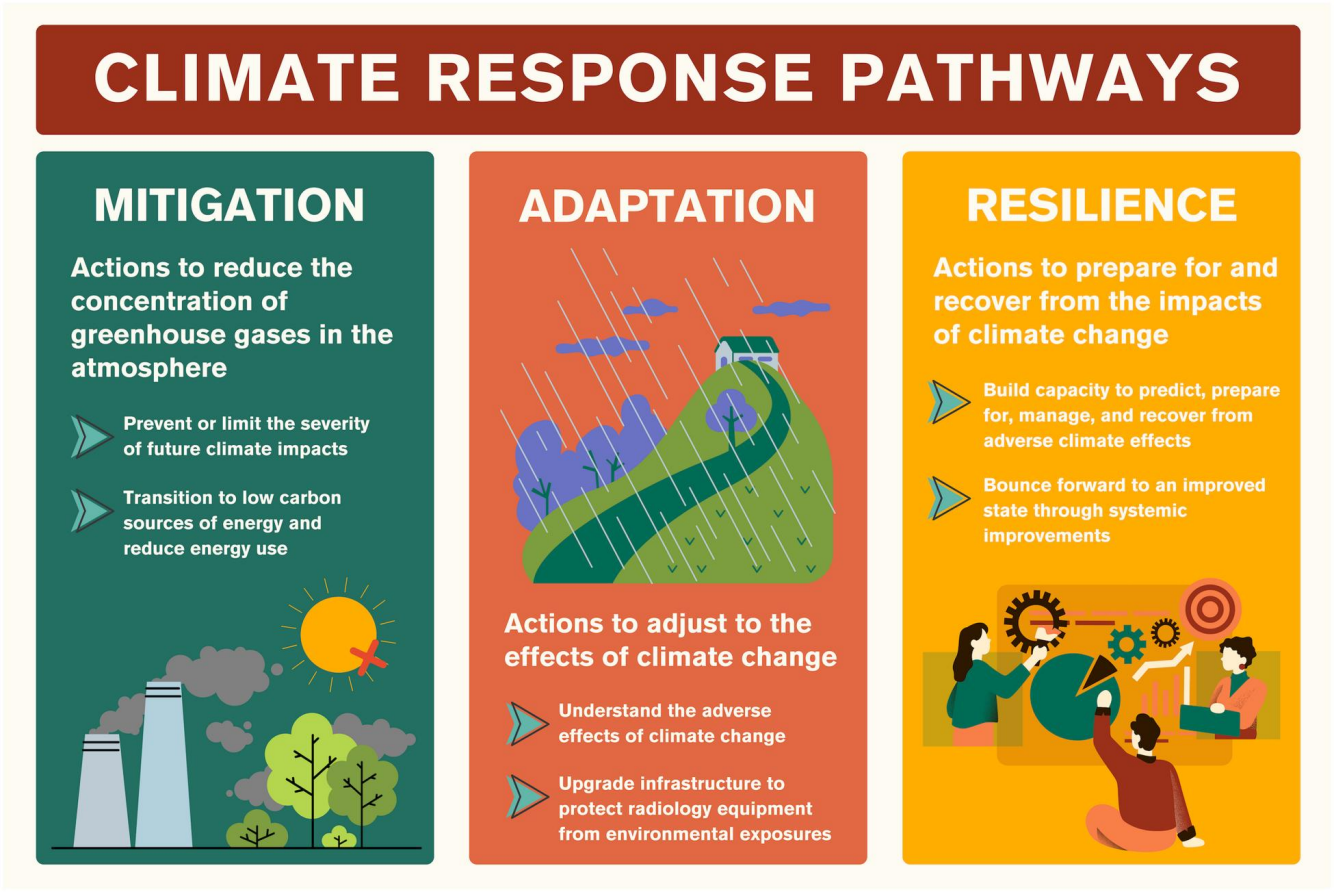
Climate response pathways include mitigation, adaptation, and resilience.

There are 3 main response pathways to address climate change: mitigation, adaptation, and resilience (Figure 1). In radiology, climate responses have largely focused on mitigation to date. Mitigation refers to all human interventions to reduce GHG emissions or to remove GHGs from the atmosphere; for example by transitioning to low carbon sources of energy and reducing energy use.^33^ The primary goal of mitigation is to prevent or limit the severity of future climate impacts, making it foundational to global efforts to achieve net-zero emissions and to limit the rise in average global temperatures.^34^

Adaptation and resilience strategies should be developed and implemented in parallel with mitigation strategies. Adaptation means adjusting to the effects of climate change and adapting to a new reality. Broadly, this includes understanding the adverse effects of climate change and acting to adjust for those effects. Examples include making changes to protect healthcare buildings and other infrastructure from environmental exposures.

Finally, resilience to climate change means preparing for the impacts of climate change and building capacity to recover quickly from those impacts. This includes the capacity to predict, manage, and prepare for the adverse effects of climate change and the ability to recover from such events. An example includes implementing a protocol to manage resources needed to meet the medical imaging needs of patients related to increased patient volumes, limited resources, and staffing shortages during climate emergencies. Enhancing resilience includes improving disaster preparedness, implementing ongoing monitoring and evaluation of sustainability practices, leveraging social and community networks, and building back after a disaster or other adverse event.

The parallel integration of mitigation, adaptation, and resilience strategies is crucial to building environmental sustainability in radiology and forms a multi-pronged approach which addresses both immediate and long-term climate impacts. Importantly, mitigation, adaptation, and resilience are all intrinsically linked. Efforts to reduce GHG emissions determine the extent to which we must adapt to the effects of climate change and the level of resilience required to recover from unavoidable climate-related events. Mitigation is of paramount importance to limit the damages of climate change. However, as climate change intensifies, our actions must extend beyond reducing GHG emissions to adopting a proactive and comprehensive approach that includes responding to environmental challenges through adaptation and resilience-building to maintain continuity of healthcare services during extreme events.^35^

### Mitigation in radiology: reducing GHG emissions

#### Energy efficiency to reduce GHG

Mitigation strategies are essential to improve sustainability in radiology and should be prioritized. Beyond the direct environmental benefits of reduced GHG emissions, mitigation strategies often yield additional co-benefits, such as cost savings and enhanced operational efficiency.^35^ Energy-related mitigation strategies to reduce GHG emissions in radiology include switching to renewable energy sources, powering down imaging equipment and computers when not in use, optimizing scanner scheduling efficiency to reduce idle time, abbreviating imaging protocols, reducing data storage by removing repeat or non-essential images, optimizing training and clinical implementation of AI tools, and refurbishing rather than replacing imaging equipment.^36–39^

Imaging equipment is energy-intensive, although GHG emissions vary by modality. In a cross-sectional analysis of a large Canadian academic multi-site radiology department, MRI and CT were the largest contributors to annual GHG emissions (41% and 34%, respectively) despite only accounting for 12% and 24% of imaging tests.^40^ Given that MRI and CT are associated with the highest proportion of GHG emissions, initial mitigation response efforts in radiology departments can be targeted to these modalities.

Powering down equipment when not in use can save substantial energy. A systematic review found that 40%-91% of the energy used by imaging devices is nonproductive, consumed while equipment is idle but left on.^41^ Idle states include ready-to-scan mode, stand-by mode, and other low-power states, including the time required for patient positioning and room turn-over between patients. A single CT scanner consumes approximately 26 226 kWh annually, with idle energy use approximately 4 times higher than energy use during active scanning.^42^ Turning off unused equipment in a radiology department could save 72 337 kWh in energy, $19 531 in cost, and 9.26 metric tons of CO_2_ emissions annually.^43^ Implementation of power-saving modes for MRI units could save 24.1 MWh of energy per scanner annually, resulting in $3373 in cost savings and 17.1 metric tons of CO_2_ emissions savings per scanner, highlighting the importance of operational strategies to minimize energy use.^44^

#### Waste reduction in radiology

**Figure 2.**
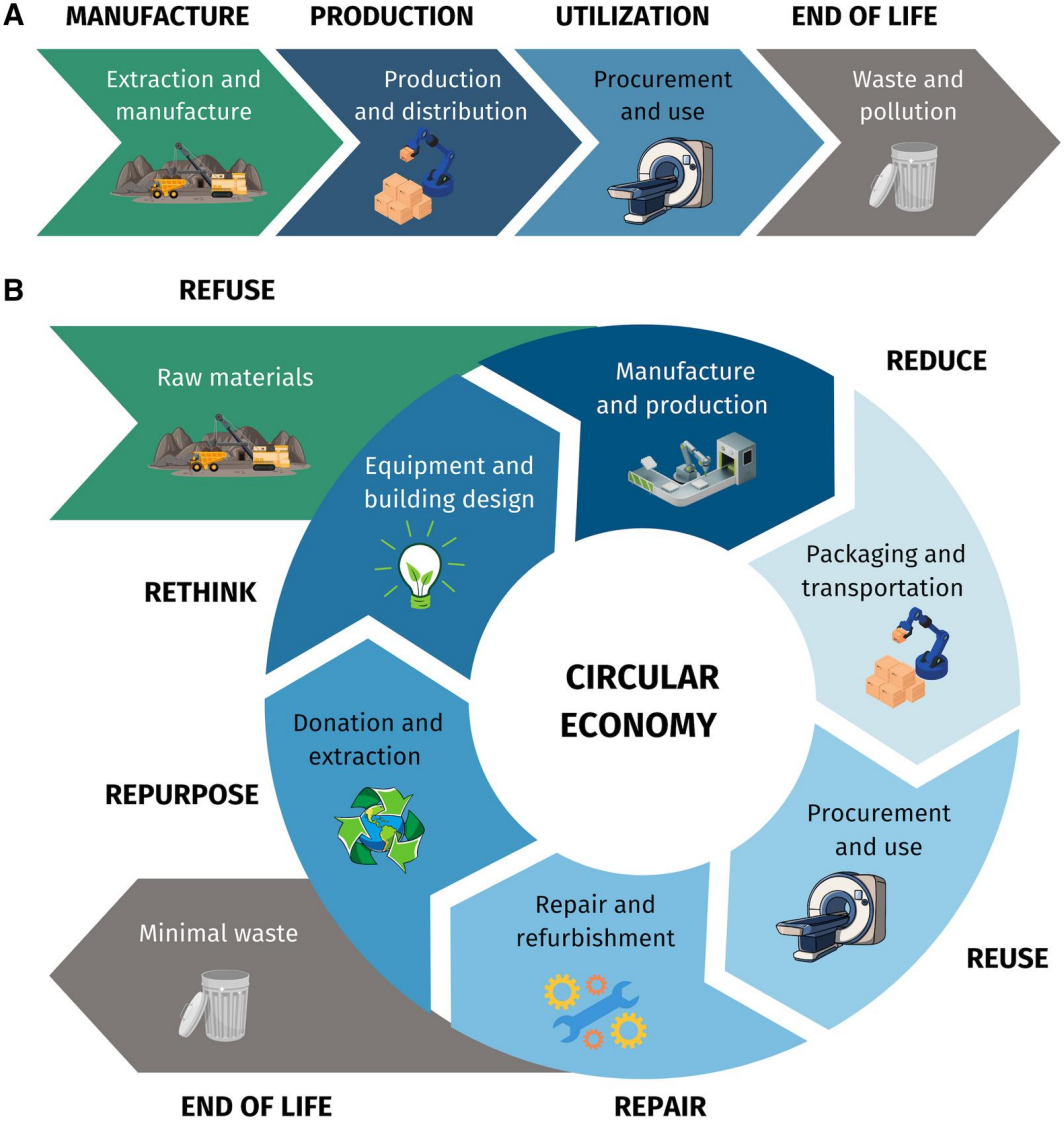
Life cycles in radiology. (A) Linear economy model, resources are extracted, manufactured, transported, used, then disposed of as wasted. (B) Circular economy, products are designed to reduce waste and maximize value over their life span through following actions: reduce, reuse, repair, repurpose, and recycle.

The lifecycle of radiological equipment, from raw material extraction to manufacturing and disposal, contributes to environmental waste. Engagement with industry is essential to move away from linear economic models (where resources are extracted, used and disposed of) toward a circular economy model that aims to extend the life cycle of products and reduce waste to a minimum through sustainable design, reuse, repair, refurbishment, and recycling (Figure 2). One solution is refurbishing older machines instead of replacing them, as recommended by the Global Diagnostic Imaging, Healthcare IT and Radiation Therapy Trade Association (DITTA) Good Refurbishment Practice.^45^ Sustainable waste management systems can improve environmental sustainability in radiology. At Imperial College Healthcare NHS Trust, the implementation of reusable sterile gowns in interventional radiology reduced medical waste, resulting in annual CO_2_ emission savings of 234 660 kg and cost savings of £27 131.^46^

### Adaptation in radiology: preparing for climate change

As climate change intensifies, the frequency and severity of natural disasters and other environmental exposures increases, creating risks to the delivery of radiology services and necessitating adaptation. Adaptation seeks to moderate or avoid harm, or even potentially exploit beneficial opportunities related to climate change. A study at Nanaimo Regional General Hospital in Canada identified rising temperatures as a significant threat to hospital systems, with the potential to overburden cooling and ventilation systems.^47^ Drier summers could also jeopardize water supplies crucial for hospital operations. Healthcare facilities are also at risk of flooding, with radiology departments at particularly high risk given that many are located in the basement or on the ground floor of buildings due to the need for shielding and the weight of the equipment. These risks, coupled with aging infrastructure, increase the likelihood of operational disruptions and compromised functionality during heat waves.^47^ As an example of an effective adaptation project, Spaulding Rehabilitation Hospital in Boston implemented design strategies to counteract flood risks where critical systems, including electrical and mechanical components, were elevated to higher levels, and the building was situated above the base flood elevation.^48^ These proactive measures address the hospital’s vulnerability due to sea level rise to ensure continuity of care during severe weather events. Grady Memorial Hospital in Atlanta experienced severe flooding due to cold-induced malfunctions in its heating and cooling systems, leading to disruptions in essential services, including the burn unit and emergency care.^49^ These incidents highlight the critical importance of ensuring airtight scanners to prevent water intrusion in order to maintain operational integrity during extreme weather events. Equally important is the implementation of robust heating and cooling systems, as well as redundancy in critical infrastructure, to ensure continuous functionality under extreme conditions. Placing equipment on higher floors or elevated platforms above anticipated flood levels reduces the risk of system failures during severe weather, safeguarding equipment functionality.

Imaging rooms should also be designed or retrofitted with waterproof materials and tightly sealed enclosures to prevent water ingress during storms or floods. This helps to protect critical equipment and reduces both downtime and the potential for damage, ensuring the continuity of services even during severe weather disruptions.^50^ Reliable backup systems are also essential to climate resilience. Uninterrupted power supplies, including redundant systems powered by renewable energy sources like solar panels or other generators, are vital during natural disasters or grid outages. These systems ensure that imaging systems remain operational, particularly during emergency situations when diagnostic services are most urgently needed.

### Resilience in radiology: responding to and moving forward from disruptions

Resilience is related but distinct from adaptation. Moving beyond adjusting to the effects of climate exposures, resilience refers to the capacity to predict, prepare for, manage, and recover from these effects. Resilience strategies are crucial for radiology departments to maintain essential services amid and beyond environmental disruptions, especially as climate change leads to an increase in patient and imaging volumes, straining systems that may already be under-resourced, understaffed, and unprepared.^51^

A vital component in building resilient radiology departments is the implementation of early warning systems.^52^ Leveraging early warning systems tailored to medical imaging will allow departments to use predictive analytics to monitor, anticipate and prepare for surges in imaging demand related to environmental exposures and other external variables. These tools can be used to guide staffing decisions in real-time, allocate workforce and other resources effectively, and schedule maintenance and other upgrades during times of expected lower volumes. In the longer term, historical imaging volume data in various settings (inpatient, emergency department, and outpatient) can be used to refine workforce and preparedness plans to ensure continuity of care.

Integrated sustainability dashboards can also provide real-time monitoring of energy use, equipment utilization, and waste generation.^35^ Long-term resilience in radiology requires systemic investments and innovation, including collaboration with manufacturers to develop energy-efficient and durable imaging equipment that is able withstand environmental extremes.^53^ Engagement with governments and policymakers is crucial to secure funding through grants, tax incentives, and public-private collaborations to support the necessary changes.

New or revealed opportunities following a crisis can potentially be taken advantage of to improve the system through broader systemic changes.^52^ This approach has been characterized as “bouncing forward” to an improved state rather than bouncing back to the pre-disruption state.^54^ As a potential opportunity, medical imaging can be used as a tool to advance our understanding of the underlying pathophysiology of climate-related health effects.^55^

## Pillars of radiology climate resilience

**Figure 3.**
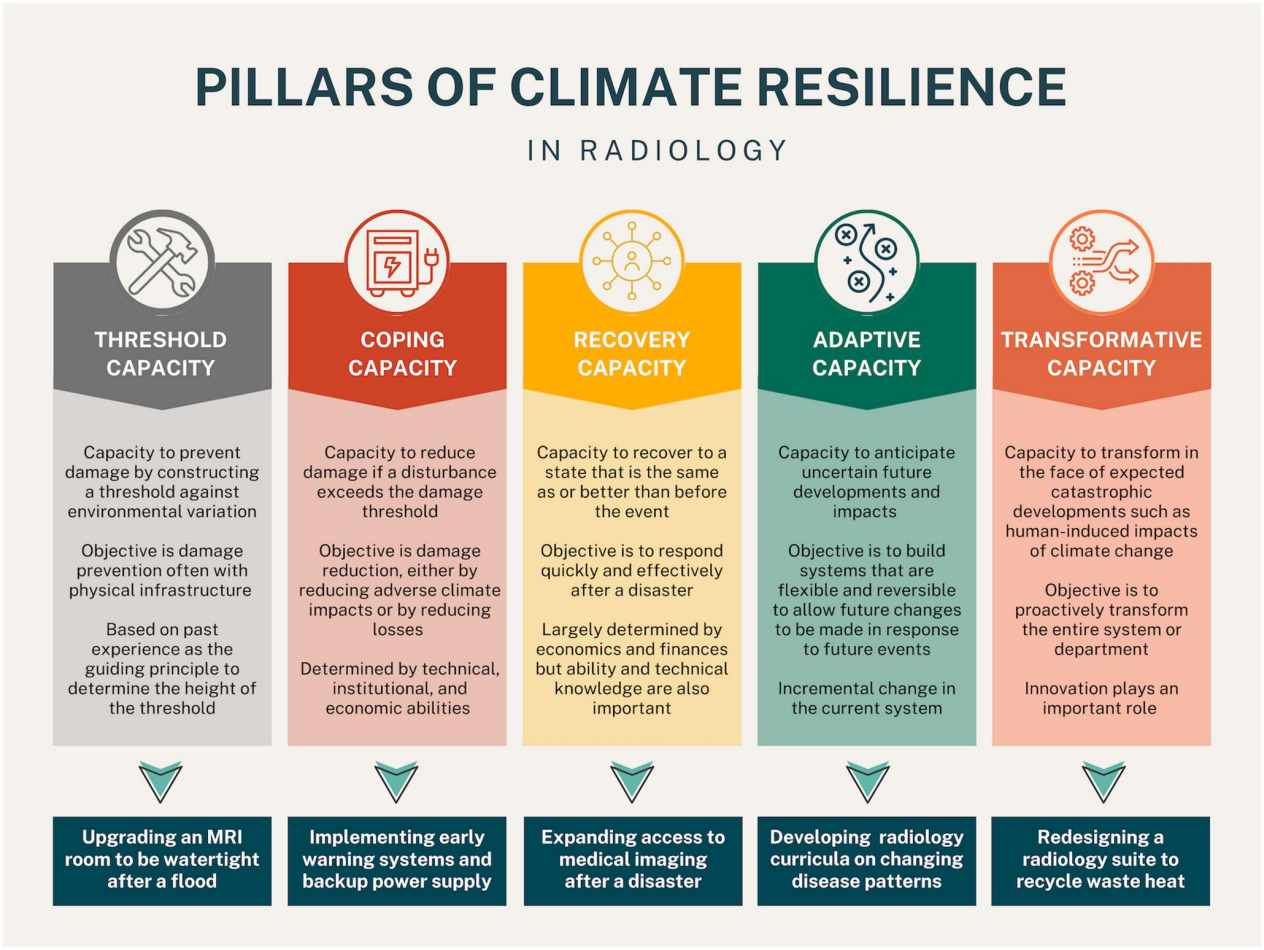
A comprehensive climate resilience strategy in radiology can be built on a 5 pilar framework which includes threshold, coping, recovery, adaptive, and transformative capacity. Collaboration with key stakeholders is needed to build climate resilient radiology systems including health system leadership teams (information technology, purchasing/procurement, security), non-radiologist providers in radiology (eg, nurses, technologists), other physicians, and medical scientists.

An overarching framework for climate resilience was proposed by De Graaf-van Dinther and Ovink.^56^ This framework consists of 5 pillars of capacity: threshold, coping, recovery, adaptive, and transformative. We have applied this 5 pillar framework to a comprehensive climate resiliency strategy in radiology (Figure 3).

### Threshold capacity

Threshold capacity is defined as the capacity to prevent damage by constructing a threshold against environmental variation. The objective is damage prevention, usually based on upgrades to physical infrastructure. The threshold varies by situation but is typically based on past experiences. For example, a radiology department that has previously experienced flooding is more likely to prioritize building scanner rooms that are watertight, although this preventative measure could be built into infrastructure planning for all scanner suites.^50^ Other infrastructure considerations include construction of healthcare and radiology facilities in locations that provide increased securities from natural disasters such as forest fires, flooding, hurricanes, tornados, and earthquakes.^57^ Opportunities for building threshold capacity in radiology also include building redundancy in information technology (IT) and communications systems to safeguard patient data and allow data access for continuity of care in the event of an environmental or other major catastrophe (such as cybersecurity incident) even if the physical department cannot be accessed.

### Coping capacity

Coping capacity refers to the capacity to reduce damage if an event exceeds the damage threshold, either by reducing the impact or reducing subsequent losses. Building coping capacity includes developing effective disaster management protocols.^56^ Coping capacity is closely related to threshold capacity, in that many of the actions to prevent damages also aid in damage reduction. In radiology, coping capacity can be developed by creating comprehensive disaster management protocols that detail steps for securing imaging equipment, protecting data and relocating staff and patients if necessary. Disaster management protocols should also include a clear communication plan and establish designated roles within the radiology department to align communication with hospital administration and emergency management teams.

### Recovery capacity

Recovery capacity is the third pillar and refers to the ability to recover to the same or better state as before the emergency. This can be expanded to encompass the concept of bouncing forward or recovery to an improved state compared to before the emergency. The objective is to respond quickly and effectively after a disaster and is largely determined by cost and finances. Recovery capacity in radiology can include actions to address health disparities such as implementing strategies to improve equitable access to imaging services and reduce the gaps in diagnostic availability across different patient populations following an adverse event. One such example is the implementation of mobile imaging units. Mobile imaging units help improve access to diagnostic imaging in rural and remote areas or in other environments which may not otherwise have access to advanced imaging.^58^^,^^59^ Further study is needed to evaluate potential trade-offs with mobile imaging with respect to reduced emissions related to patient travel but potentially higher emissions related to operation and transport of mobile imaging equipment and teams.^60^ Remote reporting, teleradiology, and remote scanning are other options to potentially build recovery capacity by reducing wait times in underserved communities and providing more timely access to care.^30^ Recovery capacity initiatives can also be adaptive; for example, robust mobile imaging and teleradiology services may prove vital to radiology care access in an era of increasing climate instability.

### Adaptive capacity

Adaptive capacity refers to the ability to anticipate uncertain future developments and impacts. As a system can’t be optimized for an unknown situation in the future, adaptive capacity needs to be built by anticipating uncertainty.^56^ The objective is to build systems that are flexible to allow future changes to be made in response to future unknown events. Radiology systems should be developed that are flexible and adaptable to future changes. Radiologists can build strong adaptive capacity by fostering flexibility in operations, adapting to new technologies and responding to changing demands. Inclusion of radiology curriculum material on climate-related health impacts—and the importance of mitigation, adaptation, and resilience—can serve as an adaptation strategy by which current and future radiologists become equipped to face climate-related events.^61^

### Transformative capacity

Transformative capacity is the fifth pillar, which is defined as the ability to transform in the face of expected catastrophic developments including the impacts of climate change. The objective is to transform an entire system or department proactively. Innovation plays an important role to create an enabling environment, strengthen stakeholder capacities, and identify and implement catalyzing interventions to build a climate-resilient culture and society.^56^ Whereas adaptive capacity involves implementing incremental changes, transformative capacity involves sizable transformative changes to entire systems that are adopted at a much larger scale or intensity.^62^ This will require substantial effort and investment to initiate and sustain these changes over time. In radiology, transformative capacity can be built through enabling green teams which can act to engage key stakeholders and promote more sustainable radiology practices locally and globally. An example would be a complete redesign of a radiology suite or department to recapture waste heat generated through powering and utilizing medical imaging equipment and recycling this to heat the room to reduce the need for additional energy use for climate-control.

## Key actions and checklist

Climate resilient and sustainable radiology practices can be supported through key actions outlined in Table 1, to increase awareness, strengthen capacity, optimize infrastructure, ensure resilient IT and communication systems, prepare and plan, manage supply chains, assess vulnerabilities, address health disparities, and measure and evaluate progress.

**Table 1.** Key actions to support climate resilient and sustainable radiology practices.

Key action	Description
Increase awareness of the relationship between climate change and radiology	Education and training including meetings, webinars, and review articles to improve climate literacy in radiology Link climate-related educational activities to continuing medical education and into radiology training programs
Strengthen capacity for longitudinal assessment and surveillance	Support research to quantify the environmental impact of delivering radiology services, imaging utilization related to climate-related environmental exposures, and measure the impact of various climate-related interventions (eg, quantifying energy reduction measures such as implementation of low-power modes on MRI unites)
Optimize building and physical infrastructure	Design buildings and physical radiology infrastructure to ensure imaging equipment is protected from water and other environmental hazards (eg, extreme temperatures, wildfires, air pollution, and power outages)
Ensure resilient IT and communication systems	Build greater redundancy in medical imaging data storage and communication systems to ensure data security and access Update disaster management protocols related to the COVID-19 pandemic or in response to other threats such as cybersecurity to address climate-related scenarios such as extreme weather events (hurricanes, tornados, flooding, etc.)
Prepare and plan	Develop disaster management and communications plans and ensure they are accessible by all relevant stakeholders Build redundancy in the radiology workforce including radiologists, technologists, and leadership teams to respond to expected and unexpected events Monitor imaging volumes and prepare for surges in imaging utilization related to climate events (eg, flooding, extreme temperatures, wildfires, air pollution)
Manage supply chains	Strengthen existing radiology supply chains through diversification of suppliers in collaboration with hospital leadership teams and vendors
Assess vulnerabilities	Assess risk in collaboration with hospital leadership and disaster management teams
Address health disparities	Prioritize access to radiology care with emphasis on underserved rural, remote, and Indigenous communities who are most affected by the effects of climate change Develop outreach programs and education to minimize the effects of climate change in vulnerable populations
Measure and evaluate progress	Employ an iterative process of learning, sharing and course correction, potentially integrated into existing quality improvement programs Measure and evaluate progress towards established goals and share successes with all team members
The radiology resiliency checklist is inspired by the Health Care Facility Climate Change Resiliency Checklist developed by the Canadian Coalition for Green HealthCare^63^	

Building on these key actions, a radiology resiliency checklist has been created to help guide radiology departments towards climate resilient and sustainable practices (key questions highlighted in Table 2 with a complete checklist available in Supplementary Materials), inspired by the Health Care Facility Climate Change Resiliency Checklist developed by the Canadian Coalition for Green HealthCare.^63^ Checklists and planning guidance were recently identified as the most useful resources to improve operational resilience.^64^ As such, it is intended that this checklist can assist radiology departments in assessing climate-related infrastructure and care-delivery vulnerabilities at both a system and facility level. Each section of the checklist addresses aspects of radiology operations in the context of climate change, ensuring that departments can maintain service delivery while minimizing their environmental impact. Outside of independent radiology facilities, many of the actions required to build sustainable radiology practices require engagement and collaboration with health system leadership and other stakeholders such as vendors, physicists, procurement, and waste management teams.^65^ It is imperative that radiologists engage multiple stakeholders and work collaboratively to switch to renewable energy sources and diversify supply chains. Representatives from multiple areas of expertise such as facilities, security, environmental services, management, community partners and local government, among others, should be included in completion of the checklist. Additionally, regular re-assessments should be completed to help facilitate ongoing improvements and adaptation of sustainable practices.

**Table 2.** Radiology resiliency checklist key questions.

Assess climate risks in radiology services
Climate change is expected to create new risks and exacerbate existing risks to health care facilities and radiology departments. Are risk assessments conducted in your radiology department to inform risk reduction activities?
Current and future climate variability and change poses risks to people and infrastructure that could affect continuity of radiology services. Does your radiology department consider climate-related hazards such as extreme outdoor temperatures, extreme storms, and risk of flooding, when conducting risk assessments?
Information about climate change and its impact on health and healthcare is growing. Do leaders in your department actively seek out opportunities to obtain information about climate-related risks that could inform risk management activities (eg, conferences, partnerships with experts)?
Emergency plans should be updated as new knowledge on climate risks becomes available. Are emergency management plans in your department updated using results from regular risk assessments?
**Determine resilience of radiology infrastructure and equipment**
Imaging equipment is vulnerable to environmental exposures such as extreme temperatures. Is your radiology facility and imaging rooms designed to withstand fluctuations in temperature?
Imaging and other equipment is susceptible to water damage. Is radiology equipment located above potential flood levels or are other protective measures in place to shield imaging equipment from water damage?
Power supplies can fail in an emergency. Does your radiology department have a backup power system to ensure continuous operation of equipment during power outages?
Back-up systems can fail if they remain unused and untested. Does your radiology department regularly assess back-up power and data storage systems to ensure reliability?
Supply chain disruptions can limit the ability to provide radiology services. Does your radiology department have a diversity of suppliers for essential supplies (eg, contrast agents)?
Climate-related disruptions can reduce health workforce availability. Does your department have a contingency plan in disaster settings including redundancy in workforce coverage (eg, radiologist and technologist)?
Reporting and communication systems may be impacted by a disaster. Does your radiology department have an emergency management protocol that specifies how radiology studies should be reported, communicated, and restored during and after a failure in the main system?
**Deliver environmentally sustainable radiology services**
Mitigation strategies are needed to reduce GHG emissions generated in the delivery of radiology services. Does your radiology department have an environmental sustainability team and action plan?
Radiology equipment is energy intensive and substantial energy is wasted in non-productive states. Is there a protocol in your department to power down imaging equipment when not in use (eg, on the weekend or overnight in an outpatient department)?
GHG emissions vary based on the source of electricity. Does your health system and radiology department use low carbon energy sources?
Substantial waste is generated in the delivery of diagnostic and interventional radiology. Does your health system and radiology department have a sustainable waste management program including accessible recycling?
Reusable supplies decrease production of waste compared to single-use supplies. Does your department prioritize reusable supplies over disposable supplies when available (eg, surgical instruments in interventional packs)?
Policies to sustainably procure medical supplies reduce medical waste, lower energy requirements, and lower costs. Does your department include environmental sustainability in procurement processes?
Climate change is associated with short-term environmental exposures (eg, extreme heat and poor air quality) that increase emergency department patient volumes. Does your department have a monitoring and response plan to identify and respond to imaging demand due to environmental exposures?
Climate change increases infectious disease risk and results in changing disease patterns. Does your radiology department provide education on changing disease patterns related to climate change?
**Measure and evaluate progress**
Data and metrics are needed to establish goals and track progress. Does your department track sustainability performance (eg, by identifying indicators of performance measurement, conducting a sustainability assessment, setting targets)?
Environmental sustainability can be integrated into existing quality assurance programs. Does your department have a process to integrate sustainability into research and quality assurance programs?
Environmental sustainability can be integrated into research programs. Does your department support or conduct research related to sustainability in radiology?
Feedback is needed to optimize sustainable policies. Does your department encourage feedback on sustainability practices and climate resilience?
Key questions in this checklist are designed to help radiology departments evaluate their current practices and identify areas for improvement in environmental sustainability and climate resilience and can be answered using the following response categories: Yes = Action completed, Somewhat = Action in progress or incomplete, No = No action planned or taken, Unknown = Status or action unknown, N/A = not applicable. A complete checklist can be downloaded in Supplementary Materials.

Abbreviation: GHG, greenhouse gas.

## Conclusion

The escalating impacts of climate change on human health and radiology systems underscore the urgent need to build climate-resilient and environmentally sustainable radiology practices.

Radiology green teams should include diverse perspectives—including radiologists, technologists, physicists, administrators, and others—and embrace a proactive approach inclusive of mitigation, adaptation and resilience strategies. By leveraging existing knowledge, implementing energy-efficient practices, optimizing resource management, and prioritizing equitable access to care, radiology practices can reduce their environment footprint while enhancing the capacity to respond to climate-related challenges while improving health equity and access to care.^66^ The 5 pillars of climate resilience provide a comprehensive framework to guide these efforts, ensuring that the immediate impacts of climate change are addressed, leading sustainable healthcare practices and bouncing forward in a stronger and more inclusive state after disruptions. Collaboration with stakeholders within and outside of radiology is needed to achieve the necessary change to foster a future where radiology contributes to both planetary health and human well-being.

## Supplementary Material

umaf014_Supplementary_Data
