# Supplementary material for: Climate resilient and environmentally sustainable radiology: a framework for implementation
Source: Radiol Adv. 2025 Apr 2;2(2):umaf014. doi: 10.1093/radadv/umaf014 (PMC12429196; doi:10.1093/radadv/umaf014)
Supplement: umaf014_Supplementary_Data [file umaf014_Supplementary_Data.zip › umaf014_Supplementary_Data.zip!/Radiology Resilience Checklist.pdf]

Radiology Resilience Checklist

Assess Climate Risks in Radiology

Climate change is expected to create new risks and exacerbate existing risks to health care facilities and radiology departments. Are risk assessments conducted in your radiology department to inform risk reduction activities?

- [ ] Yes
- [ ] Somewhat
- [ ] No
- [ ] Unknown
- [ ] N/A

Current and future climate change poses risks to people and infrastructure that could affect continuity of radiology services. Does your radiology department consider climate-related hazards such as extreme temperatures, storms, and risk of flooding, when conducting risk assessments?

- [ ] Yes
- [ ] Somewhat
- [ ] No
- [ ] Unknown
- [ ] N/A

Information about climate change and its impact on health and healthcare is growing. Do leaders in your department actively seek out opportunities to obtain information about climate-related risks that could inform risk management activities (e.g., conferences, partnerships with experts)?

- [ ] Yes
- [ ] Somewhat
- [ ] No
- [ ] Unknown
- [ ] N/A

Emergency plans should be updated as new knowledge on climate risks becomes available. Are emergency management plans in your department updated using results from regular risk assessments?

- [ ] Yes
- [ ] Somewhat
- [ ] No
- [ ] Unknown
- [ ] N/A

Determine Resilience of Radiology Infrastructure and Equipment

Imaging equipment is vulnerable to environmental exposures such as extreme temperatures. Is your radiology facility and imaging rooms designed to withstand fluctuations in temperature?

- [ ] Yes
- [ ] Somewhat
- [ ] No
- [ ] Unknown
- [ ] N/A

Imaging and other equipment is susceptible to water damage. Is radiology equipment located above potential flood levels or are other protective measures in place to shield imaging equipment from water damage?

- [ ] Yes
- [ ] Somewhat
- [ ] No
- [ ] Unknown
- [ ] N/A

Power supplies can fail in an emergency. Does your radiology department have a backup power system to ensure continuous operation of equipment during power outages?

☐ Yes ☐ Somewhat ☐ No ☐ Unknown ☐ N/A

Back-up systems can fail if they remain unused and untested. Does your radiology department regularly assess back-up power and data storage systems to ensure reliability?

☐ Yes ☐ Somewhat ☐ No ☐ Unknown ☐ N/A

Supply chain disruptions can limit the ability to provide radiology services. Does your radiology department have a diversity of suppliers for essential supplies (e.g., contrast agents)?

☐ Yes ☐ Somewhat ☐ No ☐ Unknown ☐ N/A

Climate-related disruptions can reduce health workforce availability. Does your department have a plan in disaster settings including workforce redundancy (e.g., radiologist and technologist)?

☐ Yes ☐ Somewhat ☐ No ☐ Unknown ☐ N/A

Reporting and communication systems may be impacted by a disaster. Does your radiology department have an emergency management protocol that specifies how radiology studies should be reported, communicated, and restored during and after a failure in the main system?

☐ Yes ☐ Somewhat ☐ No ☐ Unknown ☐ N/A

Deliver Environmentally Sustainable Radiology Services

Mitigation strategies are needed to reduce GHG emissions generated in the delivery of radiology services. Does your radiology department have an environmental sustainability team and plan?

☐ Yes ☐ Somewhat ☐ No ☐ Unknown ☐ N/A

Radiology equipment is energy intensive. Is there a protocol in your department to power down imaging equipment when not in use (e.g., on the weekend or overnight in an outpatient department)?

☐ Yes ☐ Somewhat ☐ No ☐ Unknown ☐ N/A

GHG emissions vary based on the source of electricity. Does your health system and radiology department use low carbon energy sources?

☐ Yes ☐ Somewhat ☐ No ☐ Unknown ☐ N/A

Substantial waste is generated in the delivery of diagnostic and interventional radiology. Does your health system and radiology department have a sustainable waste management program?

☐ Yes ☐ Somewhat ☐ No ☐ Unknown ☐ N/A

Reusable supplies produce less waste compared to single-use supplies. Does your department prioritize reusable supplies over disposable supplies (e.g., instruments in interventional packs)?

☐ Yes ☐ Somewhat ☐ No ☐ Unknown ☐ N/A

Policies to sustainably procure medical supplies reduce medical waste, energy requirements, and costs. Does your department include environmental sustainability in procurement processes?

☐ Yes ☐ Somewhat ☐ No ☐ Unknown ☐ N/A

Climate change is associated with short-term environmental exposures (e.g., heat and air pollution) that increase emergency department patient volumes. Does your department have a monitoring and response plan to identify and respond to imaging demand due to environmental exposures?

☐ Yes ☐ Somewhat ☐ No ☐ Unknown ☐ N/A

Climate change increases infectious disease risk and results in changing disease patterns. Does your radiology department provide education on changing disease patterns related to climate change?

☐ Yes ☐ Somewhat ☐ No ☐ Unknown ☐ N/A

Measure and Evaluate Progress

Data are needed to establish goals and track progress. Does your department track sustainability performance (e.g., by identifying performance indicators, conducting assessments, setting targets)?

☐ Yes ☐ Somewhat ☐ No ☐ Unknown ☐ N/A

Environmental sustainability can be integrated into existing quality assurance programs. Does your department have a process to integrate sustainability into research and quality assurance programs?

☐ Yes ☐ Somewhat ☐ No ☐ Unknown ☐ N/A

Environmental sustainability can be integrated into research programs. Does your department support or conduct research related to sustainability in radiology?

☐ Yes ☐ Somewhat ☐ No ☐ Unknown ☐ N/A

Feedback is needed to optimize sustainable policies. Does your department encourage feedback on sustainability practices and climate resilience?

☐ Yes ☐ Somewhat ☐ No ☐ Unknown ☐ N/A

This checklist is designed to help radiology departments evaluate their current practices and identify areas for improvement in environmental sustainability and climate resilience. GHG = greenhouse gas

Yes = Action completed, Somewhat = Action in progress or incomplete, No = No action planned or taken, Unknown = Status or action unknown, N/A = not applicable.
